# Diagnosis of multiple pulmonary cavernous hemangiomas via dual-layer spectral CT: A case report

**DOI:** 10.1097/MD.0000000000022495

**Published:** 2020-09-25

**Authors:** Kyungsoo Bae, Hyo Jung An, Jae Jun Jung, Ho Cheol Kim, Kyung Nyeo Jeon

**Affiliations:** a Department of Radiology, Institute of Health Sciences, Gyeongsang National University School of Medicine, Jinju; b Department of Radiology, Gyeongsang National University Changwon Hospital, Changwon; c Department of Pathology, Gyeongsang National University Changwon Hospital, Changwon, and Gyeongsang National University School of Medicine, Jinju; d Department of Thoracic Surgery, Gyeongsang National University Changwon Hospital, Changwon; e Department of Internal Medicine, Gyeongsang National University School of Medicine, Jinju, Korea.

**Keywords:** cavernous hemangioma, computed tomography, dual-energy spectral CT, lung, vascular tissue neoplasm

## Abstract

**Rationale:**

Cavernous hemangioma is a benign vascular tumor, which very rarely occurs in the lung. When appearing as multiple nodules on chest CT, this tumor can be misdiagnosed as metastatic malignancy.

**Patient concerns:**

A 72-year-old woman presented with incidentally found multiple lung nodules on chest radiograph.

**Diagnoses:**

Based on information derived from dual-layer spectral CT images, the possibility of slow flow vascular tumor such as cavernous hemangioma was suggested. A pathologic diagnosis of pulmonary cavernous hemangioma was made via video-assisted thoracoscopic biopsy.

**Interventions:**

After tissue confirmation, the patient was discharged without further intervention.

**Outcomes:**

The patient recovered without any event. Follow-up chest CT performed 6 months later showed no significant interval change in nodule size and distribution.

**Lessons:**

Material decomposition images obtained from dual energy CT can help physicians understand the character of tumor vascularity for an accurate diagnosis of pulmonary cavernous hemangioma.

## 1 Introduction

Pulmonary cavernous hemangioma (PCH) is an extremely rare benign tumor. When PCH presents as multiple nodules in both lungs, it mimics metastatic malignancy.^[1]^ Although the CT features of PCH have been reported in previous studies, particular diagnostic findings have not been described. Multi-energy applications of CT have improved the characterization of pulmonary nodules such as the differentiation between benign and malignant lesions and the assessment of tumor angiogenesis.^[2]^ Here, we describe dual-layer spectral CT findings of multiple PCHs in a 72-year-old woman with pathological correlations for a better understanding of PCH to avoid unnecessary invasive procedures.

## 2 Case report

**Figure 1:**
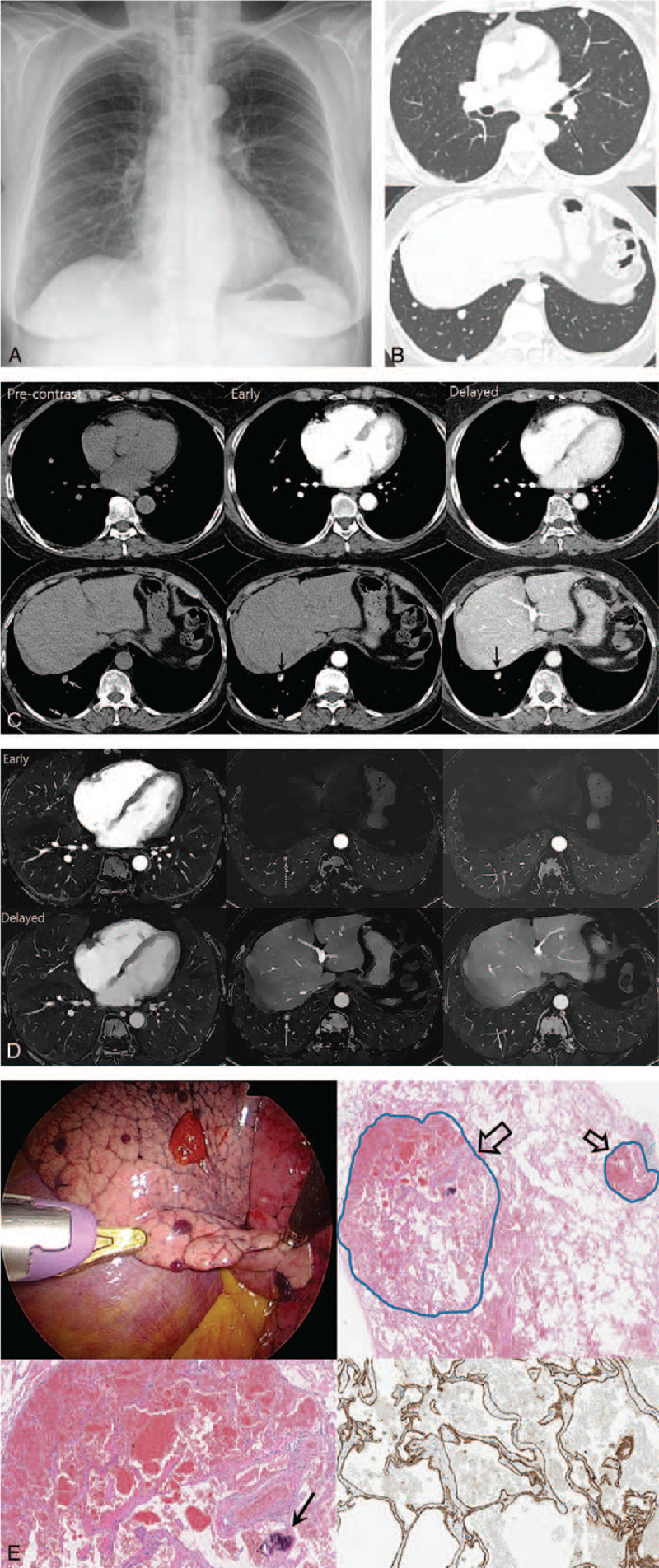
A 72-year-old female presented with an abnormal chest radiograph. (A) Chest radiograph shows multiple nodules in both lungs, especially mid and lower lung zone. (B) Chest computed tomography (CT) images in the lung window setting show multiple variable-sized nodules in both lungs. (C) Conventional chest CT images obtained using dual-layer spectral CT show multiple nodules in the right middle lobe and the right lower lobe. In pre-contrast scan (images in left column), suspicious high density foci in a few nodules (arrows) suggest calcification. In the early phase (images in middle column), a nodule in the right lower lobe shows strong enhancement (black arrow). However, other nodules show a focal peripheral dot-like enhancement (dotted arrow) or no remarkable enhancement (arrowhead). In the delayed phase (images in right column), a nodule in the right lower lobe shows extensive enhancement, whereas other nodules show no remarkable changes. (D) Iodine density images from early phase (upper row) show the contrast in each nodule (dotted arrows). Iodine density images from the delayed phase (lower row) show more extensive or centripetal filling of contrast (solid arrows). (E) Video-assisted thoracoscopic surgery (left upper) shows multiple hemorrhagic nodules in the surface of the lung. Microscopic examination (right upper) reveals well-circumscribed vascular tumors (arrows) with different sizes. Under higher magnification of the larger tumor (left lower), each vascular space shares the vein-like septate vascular walls. Calcification is noted in vessel wall (arrow). CD31 staining (right lower) shows linear, strong positive expression along the vascular endothelial cells, indicating vascular origin.

This study was approved by the Institutional Review Board of Gyeongsang National University Changwon Hospital. The patient provided written consent for publication of this case. A 72-year-old female presented with incidentally found multiple lung nodules on chest radiograph. She was a nonsmoker. The patient was treated with appendectomy due to appendicitis a month before. Other medical history was unremarkable. Chest radiograph showed multiple nodules in both lungs (Fig. 1A). Under suspicion of metastatic malignancy, a chest CT scan was performed using a dual-layer detector spectral CT scanner (IQon, Philips Healthcare). The scanning parameters were as follows: 120 kVp, 140–250 mA (reference mAs, 73), pitch of 0.609, rotation time of 0.4 seconds, 64 × 0.625 mm collimation, 1-mm slice thickness, 1-mm slice increment, and smooth filter (filter A). The nonenhanced scanning was performed first in the helical mode. Dual-phasic images were obtained after administration of 90 mL iodinated contrast material (Omnipaque 300, GE Healthcare) at a rate of 2.5 mL/s. Early-phase images were obtained using bolus tracking with the threshold set at 150 HU in aortic arch followed by a 7-second delay. Delayed phase images were obtained with a delay of 60 seconds from the beginning of contrast injection.

Chest CT showed random distribution of variable-sized nodules in both lungs (Fig. 1B). The pre-contrast scan revealed high density area in some nodules suggesting calcification (Fig. 1C). After contrast enhancement, a nodule in the right lower lobe showed focal strong enhancement in the early phase. Other nodules did not show such a strong enhancement pattern. Some nodules showed peripheral dot-like enhancing foci or no obvious enhancing area. In the delayed phase, the nodule in the right lower lobe showed more extensive enhancement, whereas other nodules showed no remarkable change. In iodine density images derived from spectral based image, the iodine distribution was noted not only in the right lower lobe but also in other nodules (Fig. 1D). The pattern of iodine distribution in the nodules reflected peripheral or smaller zone of enhancement in the early phase and centripetal filling in the delayed phase. Significant iodine concentration was detected in the nodules of both early (1.9∼5.3 mg/mL) and delayed (2.5∼6.1 mg/mL) phases. Based on information derived from conventional CT and iodine density images, the possibility of slow flow vascular tumor such as cavernous hemangioma was suggested. Hypervascular metastasis was also considered as a differential diagnostic possibility. Additional imaging studies and physical examinations did not reveal any significant lesions in other body parts.

Video-assisted thoracoscopic biopsy was performed for tissue confirmation. Grossly, multiple hemorrhagic nodules were found diffusely distributed throughout in all the lobes and pleura (Fig. 1E). Microscopic examination revealed nodules composed of large and dilated vascular channels separated from one another by scanty connective tissue stroma, and the channels were filled with blood (Fig. 1E). Calcification was observed along some of the vessel walls. Immunohistochemical staining demonstrated that the cells lining the cavernous structure stained positively for CD31, which suggested that the lesions were of vascular origin. A final pathologic diagnosis of PCH was made. The patient recovered without any event and postoperative follow-up chest CT taken 3 months later showed no significant interval change in nodule size and distribution.

## 3 Discussion

Unlike other organs such as the skin, subcutaneous tissues, and liver, cavernous hemangiomas very rarely occur as primary neoplasms in the lung.^[3]^ A recent literature review of PCH found that <20 cases have ever been reported.^[1]^ The clinical presentation of PCH varies depending on the size and number of tumors. Although some patients present with life-threatening symptoms such as severe bleeding or dyspnea, others show absent or nonspecific symptoms.^[4–6]^

PCH presents as single or multiple solid nodules on imaging studies. Multiple PCHs have been reported to mimic metastatic lesions.^[6–8]^ Metastatic nodules appear as multiple variable-sized nodules scattered in the lung and pleura. A cavernous hemangioma is an unencapsulated mass of dilated, endothelium-lined vascular channels filled with slow-flowing blood.^[6]^ In contrast to cavernous hemangiomas involving other solid organs such as the liver, a typical centripetal enhancement pattern has not been reported in PCH, hindering preoperative diagnosis.

Recent advances in CT technology have led to improved detection and characterization of nodules. In particular, dual-energy techniques enabled better characterization of nodules via material differentiation.^[2]^ The dual-layer detector spectral CT system consists of a single tube and 2-layered detectors, which simultaneously capture high- and low-energy photons during each CT examination. Because energy separation occurs on the detector, the decision to use a single- or dual-energy protocol before scanning is not necessary. Spectral information can be obtained from all examinations using a routine protocol.^[9]^ In the present study, enhancement pattern of nodules was not obvious on conventional CT images, except for a nodule in the right lower lobe because of small lesion size and high density (calcification) of the nodules. However, iodine density images derived from spectral based image clearly showed the presence and the pattern of iodine uptake in each nodule, reflecting blood supply. The pattern of iodine distribution in PCHs was similar to the contrast enhancement pattern in hepatic cavernous hemangiomas, peripheral nodular, or entire enhancement with gradual filling.^[10]^ The high-density areas observed in some nodules in the precontrast images were caused by dystrophic calcification in vessel wall as shown in pathology.

The etiology of cavernous hemangiomas is not completely understood; however, they are considered to be congenital vascular malformations, which may be attributed to genetic loss-of-function mutations.^[11]^ Management of PCH varies depending on the severity of symptoms and extent of the lesions. In a solitary PCH, surgical excision is considered as the most effective treatment. Interferon alfa-2a has been reported to be effective in treating multiple PCHs. However, because of the rarity of cases, the management of PCH remains a topic of controversy. Radiographic follow-up may play an important role in asymptomatic and stable PCH.^[12]^

We presented a rare case of multiple PCHs based on dual-layer spectral CT findings. Since percutaneous biopsy of PCH can lead to bleeding complications, a proper imaging diagnosis is important. To the best of our knowledge, this is the first report of PCH describing spectral CT findings. Despite its rarity, our informative case may help physicians understand the characteristics of the tumor based on CT and correlative pathologic findings, obviating the need for unnecessary invasive procedures.

## Author contributions

**Conceptualization:** Kyungsoo Bae, Kyung Nyeo Jeon.

**Formal analysis:** Kyungsoo Bae, Hyo Jung An.

**Investigation:** Kyungsoo Bae, Hyo Jung An.

**Methodology:** Jae Jun Jung, Ho Cheol Kim.

**Resources:** Hyo Jung An, Jae Jun Jung.

**Supervision:** Kyung Nyeo Jeon.

**Writing – original draft:** Kyungsoo Bae, Kyung Nyeo Jeon.

**Writing – review & editing:** Kyungsoo Bae, Hyo Jung An, Jae Jun Jung, Ho Cheol Kim, Kyung Nyeo Jeon.
